# Odor Characteristics of Novel Non-Canonical Terpenes

**DOI:** 10.3390/molecules27123827

**Published:** 2022-06-14

**Authors:** Svenja Sommer, Leon M. Lang, Laura Drummond, Markus Buchhaupt, Marco A. Fraatz, Holger Zorn

**Affiliations:** 1 Institute of Food Chemistry and Food Biotechnology, Justus Liebig University Giessen, Heinrich-Buff-Ring 17, 35392 Giessen, Germany; svenja.sommer@lcb.chemie.uni-giessen.de (S.S.); leon.lang@gmx.de (L.M.L.); marco.fraatz@lcb.chemie.uni-giessen.de (M.A.F.); 2 Microbial Biotechnology, DECHEMA—Forschungsinstitut, Theodor-Heuss-Allee 25, 60486 Frankfurt, Germany; laura.drummond@dechema.de (L.D.); markus.buchhaupt@dechema.de (M.B.); 3 Fraunhofer Institute for Molecular Biology and Applied Ecology, Ohlebergsweg 12, 35392 Giessen, Germany

**Keywords:** methylation, odor threshold, terpenoids, (2*E*)-decenal, terpene, flavor

## Abstract

Several non-canonical, methylated terpenes have been described as products of genetically modified *Escherichia coli* recently, and the aroma properties of 28 odor-active methylated derivatives of prenol, isoprenol, bornane, camphene, carene, citronellol, fenchol, geraniol, limonene, linalool, terpineol, and farnesol were characterized for the first time in the current study. Twelve methylated monoterpenes exhibited a particularly intense and pleasant odor and were therefore chosen for the determination of their respective odor thresholds (OTs) in comparison to their non-methylated equivalents. In addition to the determination of OTs based on the literature value for the internal standard, (2*E*)-decenal, the threshold values of the compounds with individually determined OTs of the participants were calculated. This enabled a more precise identification of the OTs. Among the non-canonical terpenes, the lowest OTs in the air were found for 2-methyllinalool (flowery, 1.8 ng L^−1^), 2-methyl-*α*-fenchol (moldy, 3.6 ng L^−1^), 2-methylgeraniol (flowery, 5.4 ng L^−1^), 2-methylcitronellol (citrus-like, 7.2 ng L^−1^), and 4-methylgeraniol (citrus-like, 16 ng L^−1^). The derivatives of geraniol, linalool, and citronellol showed very pleasant odor impressions, which could make them interesting for use as flavoring agents in the flavor and fragrance industry.

## 1. Introduction

Isoprenoids are flavor compounds, which are known for their great structural diversity and their intense odor impressions. Most isoprenoids are formed from the C_5_-prenyl pyrophosphate precursors isopentenyl pyrophosphate (IPP) and dimethylallyl pyrophosphate (DMAPP). The repeated appearance of isoprene units in terpene structures was enunciated as the isoprene rule [1]. Completed isoprenoid structures contain one or more isoprene units and differ in the occurrence of double bonds, carbonyl, carboxyl, keto, and hydroxyl groups. Aliphatic structures are named terpenes, whereas structures with functional groups are called terpenoids. Both terpene and terpenoid structures have been detected as secondary metabolites in plants, animals, and microorganisms [2]. Especially, short-chain terpenoids are relevant as aroma compounds, including hemi- (C_5_), mono- (C_10_), and sesquiterpenoids (C_15_). A common example for hemiterpenoids is prenol, which occurs, e.g., in hop or ylang-ylang flowers [3]. Monoterpenoids and monoterpenes include highly odor-active compounds such as linalool with a citrus- and lavender-like scent, thymol with a thyme-like flavor, and limonene with a fresh, orange-like odor of the (*R*)-enantiomer and a pine-like flavor of the (*S*)-enantiomer. Farnesol and (*S*)-nerolidol are examples of sesquiterpenoids that are associated with a flowery scent [2]. 

**Figure 1:**
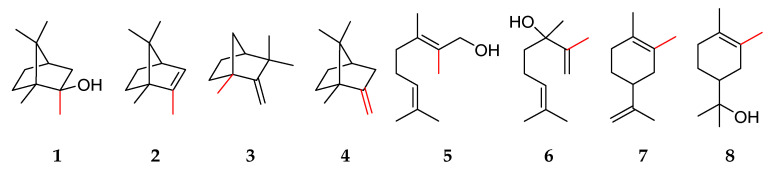
Structures of methylated terpenes, which have been identified in the environment with red-labeled bonds to the additional methyl group: 2-methylisoborneol **1**, 2-methyl-2-bornene **2**, 1-methylcamphene **3**, 2-methylenebornane **4**, 2-methylgeraniol **5**, 2-methyllinalool **6**, 2-methyllimonene **7**, and 2-methyl-*α*-terpineol **8**.

Exceptions to the isoprene rule are terpenes whose biosynthesis differs from the sequential condensation of the C_5_ units, therefore generating structures with a number of carbon atoms different from a multiple of five. These terpenes are called non-canonical terpenes. Non-canonical terpenes have been studied since the formulation of the isoprene rule itself, e.g., carotenoid degradation products. More recent studies have addressed the synthesis of non-canonical terpenes by means of methyl transferases. These enzymes catalyze the addition of methyl groups to the prenyl pyrophosphate precursors, thereby changing the final number of carbon atoms of the terpenoid structures. The methylated monoterpene 2-methylisoborneol **1** has been described, for example, in *Streptomyces* and *Actinomyces* species with an unpleasant muddy flavor and an extremely low odor threshold of 0.042 µg L^−1^ in water [4]. Furthermore, 2-methyl-2-bornene **2**, 1-methylcamphene **3**, and 2-methylenebornane **4** have been described in forest soil [5], and the methylated monoterpenes and monoterpenoids 2-methylgeraniol **5**, 2-methyllinalool **6**, 2-methyllimonene **7**, and 2-methyl-α-terpineol **8**, have been identified as products of *Nannocystis exedens* [6] (Figure 1). 

**Figure 2:**
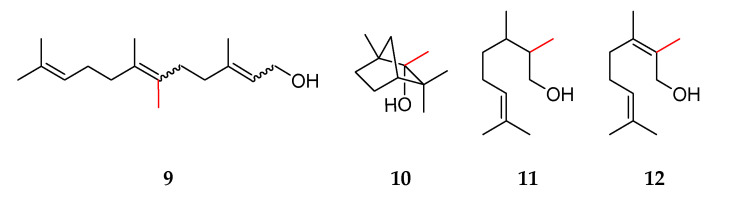
Structures of methylated terpenes described by Kschowak et al. with red-labeled bonds added to the additional methyl group: 6-methylfarnesol **9**, 2-methyl-α-fenchol **10**, 2-methylcitronellol **11**, and 2-methylnerol **12**.

Harms et al. investigated methylated sesquiterpenes such as iso-*β*-elemene and iso-germacrene, which were synthesized with a sesquiterpene synthase and showed potential as flavor compounds. Both have a citrus-like odor impression [7]. Kschowak et al. transformed *Escherichia coli* for the microbial production of novel C_11_ compounds, and Ignea et al. modified *Saccharomyces cerevisiae* to produce C_11_ terpenoids [8,9]. The genes encoding terpene synthases, including 2-methylisoborneol synthase from *Streptomyces griseus* subsp. *griseus*, 2-methylisoborneol synthase from *Streptomyces coelicolor*, 2-methylene bornane synthase from *Micromonospora olivasterospora*, and 2-methylene bornane synthase from *Pseudomononas fluorescens*, together with a geranyl pyrophosphate methyl transferase from *Streptomyces coelicolor*, were transferred. The *E. coli* strains in the above-mentioned study also included genes encoding an isopentenyl pyrophosphate (IPP) isomerase, the enzymes for the mevalonate pathway, and a geranyl pyrophosphate synthase. Kschowak et al. analyzed volatile compounds with solid-phase microextraction-gas chromatography-mass spectrometry (SPME-GC-MS) and detected several C_11_ compounds, of which 15 were identified [9]. For example, the study identified 6-methylfarnesol **9** and methylated monoterpenes such as 2-methylgeraniol **5**, 2-methyllinalool **6**, 2-methyllimonene **7**, 2-methyl-*α*-terpineol **8,** 2-methyl-*α*-fenchol **10**, 2-methylcitronellol **11**, and 2-methylnerol **12** (Figure 2). 

**Figure 3:**
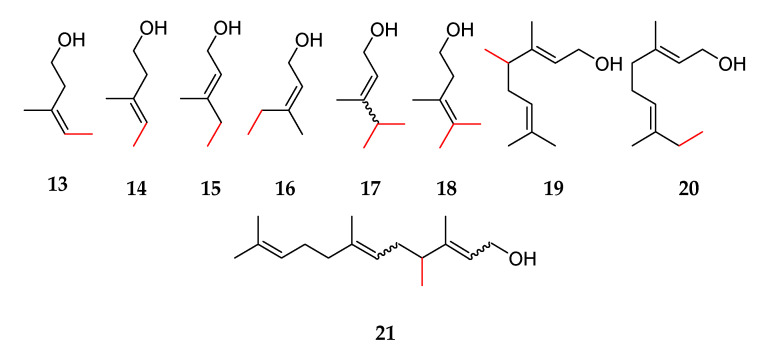
Structures of methylated terpenes described by Drummond et al. with red-labeled bonds to the additional methyl groups: (*Z*)-4-methylisoprenol **13**, (*E*)-4-methylisoprenol **14**, (*E*)-4-methylprenol **15**, (*Z*)-4-methylprenol **16**, 4,4-dimethylprenol **17**, 4,4-dimethylisoprenol **18**, 4-methylgeraniol **19**, 8-methylgeraniol **20**, and 4-methylfarnesol **21**.

Furthermore, Drummond et al. investigated the *S*-adenosyl methionine (SAM)-dependent IPP methyltransferase from *Streptomyces monomycini* and transferred the responsible genes in *E. coli* [10]. This enabled the formation of the methylated precursors (*E*)-, (*Z*)-4-methyl-IPP, 4-methyl-DMAPP, 4,4-dimethyl-IPP, and 4,4-dimethyl-DMAPP, which were released in the form of C_6_ and C_7_ alcohols. Some of these methylated precursors were accepted by a native *E. coli* farnesyl pyrophosphate (FPP) synthase, and the corresponding C_11_, C_12_, C_16_, and C_17_ compounds were formed. Examples of terpene alcohols identified in the mentioned study include (*Z*)-4-methylisoprenol **13**, (*E*)-4-methylisoprenol **14**, (*E*)- and (*Z*)-4-methylprenol **15** and **16**, 4,4-dimethylprenol **17**, 4,4-dimethylisoprenol **18**, 4-methylgeraniol **19**, 8-methylgeraniol **20**, and 4-methylfarnesol **21** (Figure 3). The biotechnological production using *E. coli* enabled the generation of a wide range of novel compounds, which have not been analyzed regarding their flavor properties so far. Due to their similarity to potent odor-active terpenes, they exhibited interesting flavor characteristics. 

Odor perception depends on the volatility of the compounds and the molecule geometry, which determines the interaction of the odotopes with the corresponding olfactory receptor proteins. Individual perceptions may differ between panelists, and the odor threshold (OT) values are not predictable so far by computational simulation [11,12]. Furthermore, fragrance impressions typically differ between the enantiomers. For instance, the mean OT of (+)-nootkatone is approximately 800-fold higher compared to that of its (–)-enantiomer (0.6–1.0 µg L^−1^ and 400–800 µg L^−1^ in water) [13]. The odor impression of (*S*)-carvone reminds one of caraway, whereas (*R*)-carvone has a minty odor [14]. The methylation of aroma compounds may also lead to aroma changes and different aroma thresholds. For example, ethyl vanillin smells vanilla-like but has an OT that is four times lower than that of vanillin [15]. 2-Nonanone has a fresh, sweetish, green, and weedy flavor, whereas 2-decanone is perceived as orange, peach-like, floral, and fatty [16,17]. 

The determination of OTs in water (OT_w_) is often performed according to Czerny et al., where the component is diluted in water and evaluated in descending concentrations in a triangle test in comparison to blanks that do not contain the aroma compound [18]. Teranishi et al. used the air to water partition coefficient to calculate the corresponding OT in air (OT_air_). According to their theory, the OT in air is proportional to the threshold in water, only depending on the relative portions of the flavor compound in the air and dissolved in water [19]. Ullrich and Grosch established a method to determine the OT in air using gas chromatography-olfactometry (GC-O) and an internal standard (IS) [20]. The standard needs to be pure, chromatographically separated from the target compound, and must have a known OT in the air. In recent studies (2*E*)-decenal became the most commonly used IS [21,22,23,24].

In this study, 28 methylated hemi-, sesqui-, and diterpenes were analyzed to determine whether they are odor-active. Especially interesting compounds were investigated by means of GC-O to determine the OT_air_ of the methylated compounds in direct comparison to those of their non-methylated analogs. To investigate whether the published OT_air_ of the IS (2*E*)-decenal is representative of the participants, the OT in water was determined for every participant and used to calculate the individual OTs in the air. 

## 2. Results

### 2.1. Determination of Purities, Response Factors, and Mass Spectra of the Methylated Compounds

As some of the synthesized non-canonical terpene standards available contained both of the respective (*E*)- and (*Z*)-isomers, their diastereomeric purities were determined. The isomers were separated by means of gas chromatography with the help of two columns of different polarity (Table 1). 

The standards of 2,4-dimethylisoprenol (line 15), 4,5-dimethylisoprenol (line 18), 4,4-dimethylprenol (line 26), 2,4-dimethylprenol (line 25), 2-methylcitronellol (line 4), 2-methylgeraniol (line 8), and 4-methylgeraniol (line 9) contained isomers that could be separated on a VF-WAXms column. The ratios of 8-methylgeraniol and 8-methylnerol (line 10) were determined on a DB-5ms column. The GC-MS spectra are listed in the Supplementary Materials (Table S1). Furthermore, the ratios of the enantiomers were measured using two different chiral columns. 2-Methyllinalool (line 21), 2-methyllimonene (line 20), and 2-methylcitronellol (line 4) represented mixtures of both enantiomers (Table 1). 4-Methylgeraniol (line 9), (*R*)-camphene, (*S*)-2-methyl-*α*-fenchol (line 7), (*R*)-α-fenchol, and (*S*)-1-methylcamphene (line 2) were found to be pure enantio. 

### 2.2. Odor Description of Methylated Hemi-, Mono-, and Sesquiterpenes

The odor impressions of methylated hemi-, mono-, and sesquiterpenes (Table 2) and of their analogous non-methylated compounds (Supplementary Materials Table S2) were described independently by 15 participants. All methylated compounds except for 6-methylfarnesol (line 6) were described with the same attributes by at least three participants. Only seven participants noted a weak odor impression for 6-methylfarnesol, whereas the others did not smell anything. The comparison of the methylated prenol derivatives with prenol and methylated isoprenol derivatives with isoprenol indicated that the position of the methyl group had an influence on the respective odor quality (Table 2 and Table S2). 

Apart from 2-methyl-α-terpineol and 2-methylenebornane (line 1, 28), which exhibited only a relatively weak odors, the methylated monoterpenes showed intense aroma impressions. Methyl-α-fenchol (line 7) was evaluated with the highest intensity, but also, 1-methylcamphene (line 2); 4-methyl-3-carene (line 3); 2-methyllimonene (line 20); 2-methyllinalool (line 21); 2-, 4-, and 8-methylgeraniol (line 8–10) had intense flavors. Therefore, these compounds were chosen for the determination of their respective OT_air_.

### 2.3. Odor Threshold of (2E)-Decenal in Water and Air

For comparison with the literature, the detection and recognition thresholds of (2*E*)-decenal were determined in triplicate. For further usage, the concentration at which at least two replicates were correctly identified was defined as the odor threshold. The participants had different detection thresholds (DT): participant 1: DT = 0.9 ± 0.3 µg L^−1^ (0.9, 0.9, and 1.8 µg L^−1^); participant 2: DT = 0.5 ± 0.1 µg L^−1^ (0.2, 0.5, and 0.9 µg L^−1^); and participant 3: DT = 1.8 ± 0.6 µg L^−1^ (0.5, 1.8, and 1.8 µg L^−1^). The recognition thresholds (RT) also differed among the participants: participant 1: RT = 3.6 ± 1.2 µg L^−1^ (1.8, 3.6, and 3.6 µg L^−1^); participant 2: RT = 0.9 ± 0.3 µg L^−1^ (0.9, 0.9, and 0.9 µg L^−1^); and participant 3: RT = 1.8 ± 0.6 µg L^−1^ (0.4, 1.8, and 1.8 µg L^−1^). The DT was used to calculate the OT_air_:

1. *OT_air,IS_* (participant 1) = 8.0 ± 2.7 ng L^−1^;
2. *OT_air,IS_* (participant 2) = 4.0 ± 1.3 ng L^−1^;
3. *OT_air,IS_* (participant 3) = 16.0 ± 5.3 ng L^−1^.

### 2.4. Odor Thresholds in Air

**Figure 4:**
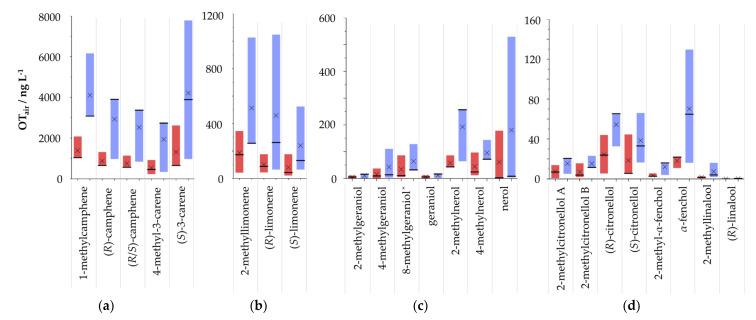
Ranges of the odor thresholds in the air from the three participants with the averages marked with a cross and the median labeled with a line for methylated and non-methylated compounds (**a**) camphene and carene derivatives; (**b**) limonene derivatives; (**c**) geraniol derivatives; (**d**) citronellol, fenchol, and linalool derivatives, according to Teranishi et al. in blue and the threshold determined with the individual determined threshold of (2*E*)-decenal in red, ^x^ = mixture of (*E*) and (*Z*) isomers.

The OT_air_ of the methylated compounds were determined in comparison to their non-methylated counterparts, which were commercially available. To each terpene mixture, (2*E*)-decenal was added as the internal standard. The D-values of the internal standard and the other compounds slightly differed between participants. The D-value is defined according to the literature as the dilution factor in which the compound can be smelled in the lowest concentration [20] (Supplementary Materials Table S3). Odor descriptions of the participants during GC-O were similar to the descriptions of the dilutions of the standards in propandiol (Table 2 and Table S4). The OT_air_ were determined with the help of the literature value of (2*E*)-decenal of 2.7 ng L^−1^ and, additionally, with the individually determined OT_air_ of each participant as described above (Figure 4) [17]. The thresholds of (2*E*)-decenal determined for the participants were higher than those reported in the literature. The OT_air_ values of 1-methylcamphene, 4-methyl-3-carene, 2-methylcitronellol, 2-methylgeraniol, 4-methylgeraniol, and 2-methyllimonene were comparable to those of their non-methylated equivalents. 8-Methylgeraniol, 2-methylnerol, and 2-methyllinalool showed higher OTs than the respective corresponding C_10_ compounds.

## 3. Discussion

The odors of 28 methylated terpenes were described. All of the studied C_6_-, C_7_-, and C_11_ compounds were perceived as aroma-active, but the methylated farnesol derivatives had only weak odors. The odor impression of a substance depends on different factors. Besides the air-to-water partition coefficient, the individual associations to known odor impressions and the interactions with the receptors in the olfactory epithelium are essential for the individual perception of the substances [25]. The descriptions of the odor characteristics varied among the participants, but the panel agreed on a set of attributes that represented the respective essential characteristics. 

Methylated aliphatic monoterpenes and methylated monoterpenoids showed the most intense odor impressions. They have molecular masses close to those of other highly odor-active compounds and high structural similarity to monoterpenes, which are well-descripted aroma compounds. Furthermore, the odor of a compound depends on the distribution between hydrophilic and lipophilic structure elements [26,27]. This matches the observation that the terpenoids had marginally lower OTs than the aliphatic terpenes. 

The human nose has approximately 430 different types of receptors [25]. The odor impression of a compound is the result of their interaction with different odotopes, which creates a pattern of signals, associated with a familiar odor. Thereby, the odor impression is dependent on the individual receptors of the nose, the association based on memories of the flavor, the health status, the age, and on other individual factors. Therefore, odor descriptions may differ between persons, and the individual thresholds can vary significantly [25,28]. 

Some of the non-canonical terpenes imparted especially interesting odor impressions. Methylcitronellol exhibited a very pleasant, intense aroma, which combined a citrus odor with intense flowery flavors. It may thus represent an interesting fragrance ingredient for cosmetics. Furthermore, the odor of methylcarene was described as fruity, sweetish, and coniferous forest-like, whereas (*S*)-carene has a resinous odor, resembling a coniferous forest. The influence of the position of the methyl group and of the stereochemistry was shown for geraniol and nerol. Geraniol with a double bound in the (*E*)-configuration has a citrus-like and flowery odor, whereas its isomer nerol, with the double bound in the (*Z*)-configuration, has a resinous, citrus-like, and flowery odor. The methylation of both compounds led to changes in the odor descriptions. The methylation of geraniol in position 8 led to a more resinous odor, the methylation in position 4 to a lemon-like odor, and the methylation in position 2 did not change the odor impression. All nerol derivatives showed a citrus-like, fruity odor but had slightly different odors. While nerol was described as resinous, flowery, citrus, and terpene-like, 2-methylnerol was sweetish, flowery, fresh, citrus, and orange-like. In contrast, 4-methylnerol was ascribed as green, fruity, flowery, and citrus-like. Furthermore, the flavor of (*R*/*S*)-methyllinalool stood out as very pleasant, similar to linalool but with notes of lemon and bergamot. Several synthetic terpenoids were developed to meet the rising need for flavoring agents. Some have intensive and highly pleasant aroma properties. For instance, the derivatives of ionone Iso E Super Plus^®^ (CAS 140194-26-9) and (–)-georgywood^®^ (CAS 828933-31-9) have odor thresholds of only few pg L^−1^ and are widely used in the cosmetics industry [29,30]. According to their odor properties, the novel geraniol and linalool derivatives could also be interesting flavoring agents, especially considering the fact that linalool and geraniol are two of the most often used flavor compounds in cosmetics, deodorants, and showering agents [31,32]. 

OTs_air_ of several monoterpenes have been determined in previous studies according to the method of Ullrich et al. [20]. Nevertheless, it is necessary to determine both odor descriptions and OTs by the same panelists to directly compare methylated and non-methylated equivalents. Overall, similar odor descriptions and OTs values as those reported in the literature have been determined in this study for monoterpenoids, but deviations were found for some compounds (Table 3). 

In particular, the OTs of the two enantiomers of citronellol were 20-fold higher than those reported by Schoenauer and Schieberle [34] but were comparable to the values determined by Elsharif and Buettner [33,34]. The individual human perception of odors varies greatly in terms of quality, threshold, pleasantness, and intensity, as it depends, e.g., on the health status, genetics, age, gender, and aroma compound [36]. Nevertheless, the panel was sensitive for all of the analyzed compounds. 

The comparison of the OTs of non-canonical terpenes with those of their canonical equivalents revealed some significant differences. While similar OTs were determined for methylated carene, nerol, limonene, and 2-methylgeraniol, the thresholds of methyllinalool and 4-methylgeraniol were higher than those of their non-methylated counterparts. Surprisingly, the OT of methylcitronellol, which had a similar odor impression as citronellol, was lower than that of citronellol.

According to Teranishi et al., the OT in the air is directly proportional to the OT_W_, only depending on the air-to-water partition coefficient [19]. Two of the panelists could detect the odor of (2*E*)-decenal during GC-O in all dilutions up to 1:64 and one participant up to 1:128. The panelist who perceived the odor up to 1:128 dilution also had the lowest OT_W_ (0.5 ± 0.1 µg L^−1^). The OT in water of the two participants who detected the odor until a dilution of 1:64 were 0.9 ± 0.3 and 1.8 ± 0.6 µg L^−1^ in water. The thresholds determined in water of all participants differed from the literature threshold of (2*E*)-decenal (0.3 µg L^−1^) [19]. The lowest concentration at which the participants could detect the odor during GC-O was proportional to the individual OT in water. Using the threshold from the literature leads to a less precise determination of the OTs by GC-O as the same value was taken for all participants even if their OTs differed and they perceived the odor until different dilution steps. Furthermore, the sensitivity of the human nose can also be different for different compounds [36]. Accordingly, the determination of individual OTs of the IS is as important as the individual determination of the thresholds of the new compounds. 

For some compounds, the OT adopted for the internal standard did not significantly influence the calculations of the OTs. However, there was a strong influence observed for some of the evaluated compounds. For instance, the standard deviations calculated for 2-methylcitronellol, citronellol, 8-methylgeraniol, 4-methylnerol, and geraniol were multiple-fold smaller, with the individually determined OTs compared to those using the fixed literature OT. On the other hand, for *α*-fenchol, (*R*)-limonene, and 2-methyllinalool, the standard deviations were higher with the individually determined thresholds. Overall, the method proposed here is more precise, and the calculated thresholds of the analyzed compounds were higher when the individually determined OTs of (2*E*)-decenal were used for calculation. The individual OTs of an IS may be used to determine OTs for additional ISs, which could be more similar to the analyzed compounds, as suggested by Ullrich and Grosch [20]. If the air-to-water partition coefficient or the Henry constant are known, every substance could be used to calculate the threshold in the air. 

## 4. Materials and Methods

### 4.1. Chemicals

Pure solvents were purchased: 1,2-propandiol (99,5%) from Carl Roth GmbH & Co. KG (Karlsruhe, Germany), dichloromethane (≥99.9%) and ethanol (≥99,9%) from Chemsolute (Renningen, Germany), and methanol (≥99.8%) from J. T. Baker (Deventer, The Netherlands). Authentic standards of non-methylated terpenoids were obtained from commercial sources: geraniol (99%) and (*R*)-(–)-linalool (95%) from Acros Organics B.V.B.A (Fair Lawn, NJ, USA); (+)-fenchol (96%), (*E*,*E*)-farnesol (97%), isoprenol (97%), and (*S*)-(–)-limonene (97%) from Alfa Aesar (Kandel, Germany); linalool (97%), (+)-camphene (80%), (*R*)-(+)-citronellol (97%), (*R*)-(+)-limonene (97%), and (–)-*α*-terpineol (≥96%) from Sigma Aldrich (St. Louis, MO, USA); and (±)-camphene (>78.0%), (+)-3-caren (>90%), (*S*)*-*(–)-citronellol (>98%), nerol (>98.0%), and prenol (>98.0%) from TCI Deutschland GmbH (Eschborn, Germany). The internal standard (2*E*)-decenal (95%) was obtained from Alfa Aesar. 

**Figure 5:**
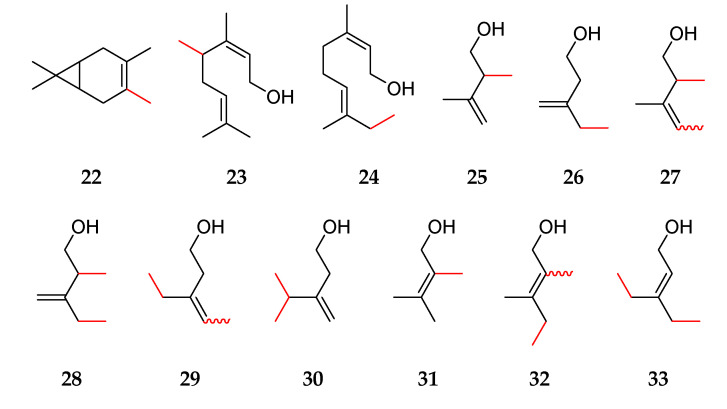
Structures of methylated terpenes with red-labeled bonds to the additional methyl groups: 4-methyl-3-carene **22**, 4-methylnerol **23**, 8-methylnerol **24**, 2-methylisoprenol **25**, 5-methylisoprenol **26**, 2,4-dimethylisoprenol **27**, 2,5-dimethylisoprenol **28**, 4,5-dimethylisoprenol **29**, 5,5-dimethylisoprenol **30**, 2-methylprenol **31**, 2,4-dimethylprenol **32**, and 4,5-dimethylprenol **33**.

Methylated terpenes were synthesized by Enamine Ltd. (Riga, Latvia): 2-methylene bornane **4** (95%), 1-methylcamphene **3** (95%), 4-methyl-3-carene **22** (95%), 2-methylcitronellol **11** (95%), 4-methylfarnesol **21** (95%), 6-methylfarnesol **9** (95%), (1*S*)-2-methyl-*α*-fenchol **10** (95%), (*E*/*Z*)-mixture of 2-methylgeraniol **5** and 2-methylnerol **12** (95%), (*E*/*Z*)-mixture of 4-methylgeraniol **19** and 4-methylnerol **23** (95%), (*E*/*Z*)-mixture of 8-methylgeraniol **20** and 8-methylnerol **24** (95%), 2-methyllimonene **7** (95%), 2-methyllinalool **6** (95%), 2-methylisoprenol **25** (95%), 5-methylisoprenol **26** (95%), 2,4-dimethylisoprenol **27** (95%), 2,5-dimethylisoprenol **28** (99%), 4,4-dimethylisoprenol **18** (95%), 4,5-dimethylisoprenol **29** (99%), 5,5-dimethylisoprenol **30** (95%), 2-methylprenol **31** (95%), (*Z*)-4-methylprenol **15** (≥95%), (*E*)-4-methylprenol **16** (≥95%), 2,4-dimethylprenol **32** (95%), 4,4-dimethylprenol **17** (95%), 4,5-dimethylprenol **33** (95%), and 2-methyl-*α*-terpineol **8** (95%) (Figure 1, Figure 2, Figure 3 and Figure 5). (*Z*)-4-Methylisoprenol **13** (≥95%) and (*E*)-4-methylisoprenol **14** (≥95%) were purchased from AKos GmbH (Lörrach, Germany).

### 4.2. Sensory Analysis

Fifteen participants (eight women, seven men, 23–34 years) described odors of the pure compounds, dissolved in 1,2-propandiol, freely. Therefore, 1 µL or 0.95 mg were dissolved in 200 µL of 1,2-propandiol, and 4 µL of the solutions were placed on a filter paper strip and marked with a three-digit code. The intensity of each odor impression was evaluated from 0 (no odor) to 5 (very intense odor). 

### 4.3. Gas Chromatographic Analysis

The retention indices of the analytes on a polar column and their respective mass spectra were measured with a gas chromatography-mass spectrometry (GC-MS) system. An Agilent 7890A GC, together with an Agilent 7000B MS triple Quad (Agilent Technologies, Santa Clara, CA, USA) equipped with a VF-WAXms column (30 m, ID 250 µm, film thickness 0.25 µm; Agilent Technologies), were used. Helium 5.0 (Nipon Gasses GmbH, Hürth, Germany) was used as the carrier gas with a constant flow rate of 1.56 mL min^−1^. The gas flow was split 1:1 between the MS and the ODP port (ODP 3, GERSTEL GmbH & Co. KG, Mülheim a.d., Ruhr, Germany). One microliter of the sample solution was injected in a splitless liner at 250 °C. The oven was heated to 40 °C (3 min)/5 °C min^−1^/240 °C (12 min). The mass spectrometer was equipped with an electron ionization source (230 °C, 70 eV) and operated in scan mode (*m*/*z* 33–300). 

The retention indices on the non-polar DB-5ms column (30 m–320 µm–0.25 µm) were determined by means of a gas chromatography-flame ionization detector system (GC-FID) with a 7890 A GC (Agilent technologies). Measurements were performed as indicated above, except for the following parameters: the carrier gas was hydrogen 5.0 (Nipon Gasses GmbH) with a flow rate of 2 mL min^−1^, and the oven was heated with the same ramp to 320 °C (12 min). The FID was heated at 250 °C. Retention indices (RI) were calculated according to van den Dool and Kraatz [37].

Chiral analyses were performed using a GC-FID 6890A (Agilent Technologies) equipped with a Hydrodex *β*-6-TBDM column (25 m–250 µm, Macherey Nagel GmbH & Co. KG, Düren, Germany). One microliter was injected in a splitless liner, which was heated to 250 °C. The GC oven was heated at 80 °C (0 min)/2 °C min^−1^ to 150 °C/20 °C min^−1^ to 250 °C (5 min). The pressure was constant at 0.8 bar, with nitrogen as the carrier gas. 

Enantiomeric distribution of methylcitronellol was measured with a Shimadzu GC-MS QP2010 SE on an Astec CHIRALDEX *β*-DM (Supelco Inc., Bellefonte, PA, USA; 30 m–250 µm, and 0.12 µm). The injection volume was 1 µL, the column flow was 0.84 mL min^−1^, and helium was used as the carrier gas. The source temperature was 180 °C, and molecular masses were scanned from *m*/*z* 40–400. The oven was heated with 40 °C (6 min)/5 °C min^−1^ to 120 °C (40 min)/10 °C min^−1^ and to 180 °C (1 min). 

The ratios of (*E*) and (*Z*) isomers were calculated with Formula (1).

(1) $$r_{EZ}=\frac{peak\;area\;\left(isomer\right)}{peak\;area\;\left(E\right)+peak\;area\;\left(Z\right)}$$

The ratios of (*R*) and (*S*) enantiomers and (*R_RS_*) were determined according to Formula (2). Enantiomeric ratios of methylcitronellol were calculated with Formula (3) because the compounds could not be baseline-separated. Enantiomeric excess (*ee*) was calculated with Formula (4).

(2) $$r_{RS}=\frac{peak\;area\;\left(enantiomer\right)}{peak\;area\;\left(R\right)+peak\;area\;\left(S\right)}$$

(3) $$r_{RS,Citronellol}=\frac{peak\;hight\;\left(enantiomer\right)}{peak\;hight\left(R\right)+peak\;hight\;\left(S\right)}$$

(4) $$ee=\frac{\left|r_{R}\right.\;\left.-r_{S}\right|}{r_{R}+r_{S}}\cdot 100\%$$

### 4.4. Odor Thresholds of the Internal Standard (2E)-Decenal

The odor threshold of the internal standard (2*E*)-decenal in water (*OT_W,IS_*) was determined in pure water, as described by Hammer et al. [21]. The initial concentration was 38 µg L^−1^, and the solution was diluted 1:2 (*v*/*v*) nine times. The tests were carried out in triplicate by each of the three participants, who also performed the GC-O analyses.

The corresponding individual odor threshold in the air of the internal standard (*OT_air,IS_*) was then calculated for each participant according to Teranishi et al. with the help of the previously determined odor threshold in water (*OT_W,IS_*) and the air-to-water partition coefficient *K_W_* with Formula (5) [19].

(5) $$OT_{air,IS}=OT_{W,IS}\cdot \;K_{W}=OT_{W,IS}\cdot \frac{c_{air}}{c_{W}}$$

### 4.5. Odor Thresholds in Air

The analyses were done according to Hammer et al., with adapted oven temperature ramps [21]. The compounds were dissolved in methanol, and the concentrations were chosen individually according to their respective aroma potency. The compounds were analyzed in four mixtures (Table 4).

Mixtures 1–3 were measured with a temperature program of 40 °C (5 min)/5 °C min^−1^ to 160 °C (0 min)/20 °C min^−1^ to 240 °C (4 min) and mixture 4 with 40 °C (5 min)/5 °C min^−1^ to 140 °C (2 min)/5 °C min^−1^ to 160 °C (0 min)/20 °C min^−1^ to 240 °C (4 min). The mixtures were successively diluted 1:2 (*v*/*v*) with methanol. The determination of the OT_air_ by GC-O was done by one man and two women, which were 24–29 years old. Samples were analyzed in a random order, and each participant noted the odor individually.

The OT_air_ of the analyzed compound X (*OT_air,X_*) was calculated with the OT_air_ of the IS (2*E*)-decenal (*OT_air,IS_*), the initial concentration of the analyzed compound *c_x_*, the D-value of the IS *D_IS_*, the initial concentration of the IS (*c_IS_*), and the D-values of the analyzed compound *D_x_* and of the IS *D_IS_* with Formula (6).

(6) $$OT_{air,x}=\frac{OT_{air,IS}\cdot c_{x}\cdot D_{IS}}{c_{IS}\cdot D_{x}}$$

## 5. Conclusions

This study characterized 28 novel methylated terpenes regarding their odor and their chemical characteristics, including mass spectra and retention indices on two columns of different polarities. Thirteen of the evaluated non-canonical terpenes showed intense aroma impressions, and the OTs were determined for the first time in comparison to those of eleven reference terpenes. Individual determination of the OTs of the IS enabled us to determine the thresholds more precisely and to expand the options for an internal standard.

## 6. Patents

Parts of this study are included in a European Patent “The use of non-canonical terpenes or terpenoids as aroma chemicals”, Sommer, S., Fraatz, M.A., Zorn, H. (19 May 2022, EP 22174377.6). 

## Figures and Tables

**Table 1:** (**a**) Retention indices on a polar VF-WAXms column and a nonpolar DB-5ms column, ratios of (*E*/*Z*) isomers, ratios of (*R*)- and (*S*)-enantiomers, and enantiomeric excess (*ee*). (**b**) Retention indices on a polar VF-WAXms column and a nonpolar DB-5ms column, ratios of (*E*/*Z*) isomers, ratios of (*R*)- and (*S*)-enantiomers, and enantiomeric excess (*ee*).

**(a)**				
	**Compound**	**RI**	**Ratios/%**	***ee*/%**
1	2-methylenebornane **4**	VF-WAXms: 1120	-	-
		DB-5ms: 1017	-	
2	(*S*)-1-methylcamphene **3**	VF-WAXms: 1075	-	100%
		DB-5ms: 985	(*R*/*S*): 0/100	
3	4-methyl-3-carene **22**	VF-WAXms: 1229	-	-
		DB-5ms: 1091	-	
4	2-methylcitronellol **11**	VF-WAXms: 1824, 1834	(*E*/*Z*): 64/36 ^#^	30%
		DB-5ms: 1301, 1305	(*R*/*S*): 65 ^+^/35 ^+,#^	
5	4-methylfarnesol **21**	VF-WAXms: 2348	-	-
		DB-5ms: 1749	-	
6	6-methylfarnesol **9**	VF-WAXms: 2380, 2430	-	-
		DB-5ms: 1790	-	
7	(*S*)-2-methyl-*α*-fenchol **10**	VF-WAXms: 1606	-	100%
		DB-5ms: 1199	(*R*/*S*): 0/100	
8	2-methylgeraniol **5**/2-methylnerol **12**	VF-WAXms: 1843 *^Z^*^,^*, 1884 *^E^*	(*E*/*Z*): 50/50	-
		DB-5ms: 1299 *^Z^*^,^*, 1317 *^E^*	-	
9	4-methylgeraniol **19**/4-methylnerol **23**	VF-WAXms: 1807 *^Z^*^,^*, 1857 *^E^*	(*E*/*Z*): 8/14	100%
		DB-5ms: 1265 *^Z^*^,^*, 1293 *^E^*	(*R*/*S*): 100/0 ^#^	
10	8-methylgeraniol **20**/8-methylnerol **24**	VF-WAXms: 1919 *^Z^*^,^*, 1923 *^E^*	(*E*/*Z*): 75/25	-
		DB-5ms: 1334 *^Z^*^,^*, 1341 *^E^*	-	
11	2-methylisoprenol **25**	VF-WAXms: 1283	-	-
		DB-5ms: 812	-	
12	5-methylisoprenol **26**	VF-WAXms: 1348	-	-
		DB-5ms: 842	-	
13	(*E*)-4-methylisoprenol **14**	VF-WAXms: 1363	-	-
		DB-5ms: 861	-	
14	(*Z*)-4-methylisoprenol **13**	VF-WAXms: 1374	-	-
		DB-5ms: 856	-	
**(b)**				
	**Compound**	**RI**	**Ratios/%**	***ee*/%**
15	2,4-dimethylisoprenol **27**	VF-WAXms: 1393, 1401	(*E*/*Z*): 86/14 ^#^	-
		DB-5ms: 916, 924	-	
16	2,5-dimethylisoprenol **28**	VF-WAXms: 1378	-	-
		DB-5ms: 904	-	
17	4,4-dimethylisoprenol **18**	VF-WAXms: 1477	-	-
		DB-5ms: 958	-	
18	4,5-dimethylisoprenol **29**	VF-WAXms: 1441, 1467	(*E*/*Z*): 63/37 ^#^	-
		DB-5ms: 944, 949	-	
19	5,5-dimethylisoprenol **30**	VF-WAXms: 1400	-	-
		DB-5ms: 906	-	
20	2-methyllimonene **7**	VF-WAXms: 1299	-	0%
		DB-5ms: 1122	(*R*/*S*): 50/50	
21	2-methyllinalool **6**	VF-WAXms: 1620	-	0%
		DB-5ms: 1190	(*R*/*S*): 50/50	
22	2-methylprenol **31**	VF-WAXms: 1407	-	-
		DB-5ms: 877	-	
23	(*Z*)-4-methylprenol **16**	VF-WAXms: 1393	-	-
		DB-5ms: 866	-	
24	(*E*)-4-methylprenol **15**	VF-WAXms: 1416	-	-
		DB-5ms: 881	-	
25	2,4-dimethylprenol **32**	VF-WAXms: 1467, 1478	(*E*/*Z*): 50/50	-
		DB-5ms: 951, 956	-	
26	4,4-dimethylprenol **17**	VF-WAXms: 1448, 1470	(*E*/*Z*): 13/87 ^#^	-
		DB-5ms: 929, 944	-	
27	4,5-dimethylprenol **33**	VF-WAXms: 1487	-	-
		DB-5ms: 959	-	
28	2-methyl-*α*-terpineol **8**	VF-WAXms: 1785	-	-
		DB-5ms: 1286	-	

* = (*Z*)-isomer of methyl-geraniol is called methyl-nerol, ^+^ = enantiomeric ratio of both (*E*/*Z*) isomers; and ^#^ = only relative portions are available, no assignment to *(R*) or (*S*) and (*E*) or (*Z*); ratios are listed according to their retention times on VF-WAXms for (*E*/*Z*) or chiral column for (*R*/*S*).

**Table 2:** Odor descriptions of pure methylated hemi-, mono-, and sesquiterpenes, which were given by at least three participants, with the number of mentions in parentheses (*n* = 15).

	Substances	Odor Impression	Intensity
1	2-methylenebornane **4**	earthy (4), coniferous forest (3), resinous (3)	0.9 ± 0.8
2	(*S*)-1-methylcamphene **3**	resinous (10), coniferous forest (9), woody (3), fruity (3)	3.3 ± 1.0
3	4-methyl-3-carene **22**	fruity (7), coniferous forest (7), resinous (6), sweetish (4), pepper (4), mint (3), citrus (3)	3.5 ± 0.8
4	2-methylcitronellol **11**	flowery (8), citrus (6), rose (4), sweetish (3), ethereal (3), fruity (3)	3.9 ± 0.8
5	4-methylfarnesol **21**	citrus (5), resinous (5), green (3)	1.7 ± 1.0
6	6-methylfarnesol **9**	-^#^	0.7 ± 0.8
7	(*S*)-2-methyl-*α*-fenchol **10**	earthy (13), moldy (9), moss (3), beetroot (3)	4.8 ± 0.4
8	2-methylgeraniol **5**/2-methylnerol **12** *	flowery (8), citrus (5), resinous (4), rose (4), sweetish (3)	2.9 ± 1.4
9	4-methylgeraniol **19**/4-methylnerol **23** *	citrus (8), lemon (3), lemon peel (3)	3.7 ± 0.8
10	8-methylgeraniol **20**/8-methylnerol **24** *	flowery (8), resinous (6), sweetish (5), citrus (4), varnish (4)	2.5 ± 0.8
11	2-methylisoprenol **25**	resinous (5), sweetish (3), coniferous forest (3), fruity (3)	1.6 ± 1.2
12	(*E*)-4-methylisoprenol **14**	green (8), grass (4), herbal (4), coniferous forest (3), apple (3)	2.5 ± 0.8
13	(*Z*)-4-methylisoprenol **13**	flowery (9), green (6), fruity (5), apple (4)	3.1 ± 1.0
14	5-methylisoprenol **26**	pungent (6), solvent (6), glue (4), varnish (3)	4.6 ± 0.8
15	2,4-dimethylisoprenol **27**	coniferous forest (5), green (4), resinous (4)	2.5 ± 0.7
16	2,5-dimethylisoprenol **28**	resinous (9), coniferous forest (7), mint (3), green (3), varnish (3)	3.2 ± 1.2
17	4,4-dimethylisoprenol **18**	green (6), citrus (4), flowery (3), soapy (3), grass (3)	2.5 ± 1.1
18	4,5-dimethylisoprenol **29**	resinous (4), woody (3), coniferous forest (3)	1.5 ± 1.1
19	5,5-dimethylisoprenol **30**	coniferous forest (10), resinous (8), woody (3)	3.4 ± 1.1
20	2-methyllimonene **7**	resinous (6), terpene (4), mushroom (4)	3.7 ± 0.8
21	2-methyllinalool **6**	flowery (11), citrus (9), sweetish (8), fruity (6), bergamot (5), blueberry (4), lavender (3)	3.7 ± 0.5
22	2-methylprenol **31**	plastic (3), terpene-like (3)	1.5 ± 0.9
23	(*Z*)-4-methylprenol **16**	plastic (3), terpene-like (3), chemical (3)	1.7 ± 0.8
24	(*E*)-4-methylprenol **15**	sweetish (5), flowery (5), green (5), citrus (3), fresh (3), resinous (3)	2.3 ± 1.4
25	2,4-dimethylprenol **32**	resinous (4), woody (3), coniferous forest (3), glue (3), sweetish (3)	2.8 ± 1.3
26	4,4-dimethylprenol **17**	sweetish (6), fruity (3)	2.1 ± 1.3
27	4,5-dimethylprenol **33**	woody (6), resinous (3), plastic (3)	3.1 ± 1.1
28	2-methyl-*α*-terpineol **8**	sweetish (4), green (3)	1.1 ± 1.1

* Mixture of (*E*)- and (*Z*)-isomers. ^#^ No impression was named by ≥3 participants.

**Table 3:** Comparison of odor thresholds in the air (OT) reported in the literature and determined in this study.

	Compound	OT (Literature)/ng L^−1^	OT (This Study)/ng L^−1^
1	(*R*/*S*)-citronellol	11 [33]	n.d.
2	(*R*)-citronellol	1.1 [34]	24 ± 19
3	(*S*)-citronellol	0.57 [34]	19 ± 23
4	geraniol	0.067 [34], 11.5 [33]	5.7 ± 5.4
5	nerol	61 [34], 68 [35]	61 ± 100
6	(*R*)-limonene	135 [35]	100 ± 67
7	(*S*)-limonene	270 [35]	81 ± 84
8	(*R*/*S*)-linalool	0.26 [34], 3.2 [33]	n.d.
9	(*R*)-linalool	0.036 [35]	0.098 ± 0.064

n.d. = not determined.

**Table 4:** Composition of the four mixtures used for the GC-O analysis for determination of the odor thresholds in the air.

	Compounds
Mixture 1	*(R*)-camphene (578 mg L^−1^), (*S*)-limonene (310 mg L^−1^), 2-methyllimonene (304 mg L^−1^), (*R*)-linalool (38.0 mg L^−1^), 2-methylfenchol (38.0 mg L^−1^), (2*E*)-decenal (38.0 mg L^−1^), (*R*)-citronellol (77.4 mg L^−1^), geraniol (39.5 mg L^−1^), and 8-methylgeraniol ((*E*/*Z*)-mixture, 152 mg L^−1^)
Mixture 2	4-methyl-3-carene (405 mg L^−1^), (*R*)-*α*-fenchol (38.4 mg L^−1^), 2-methyllinalool (38.0 mg L^−1^), (2*E*)-decenal (38.0 mg L^−1^), (*S*)-citronellol (78.3 mg L^−1^), and 2-methylgeraniol ((*E*/*Z*)-mixture, 152 mg L^−1^)
Mixture 3	(*R*/*S*)-camphene (500 mg L^−1^), (*S*)-3-carene (576 mg L^−1^), (*R*)-limonene (621 mg L^−1^), (2*E*)-decenal (38.0 mg L^−1^), nerol (78.4 mg L^−1^), and 4-methylgeraniol ((*E*/*Z*)-mixture, 152 mg L^−1^)
Mixture 4	(*S*)-1-methylcamphene (456 mg L^−1^), (2*E*)-decenal (38.0 mg L^−1^), and 2-methylcitronellol ((*E*/*Z*)-mixture, 152 mg L^−1^)

## Data Availability

The data presented in this study are available on request from the corresponding author. The data are not publicity available due to the large data set.

## Supplementary Materials

The following supporting information can be downloaded at https://www.mdpi.com/article/10.3390/molecules27123827/s1. Table S1: Gas chromatography-mass spectra of unpublished compounds with the relative intensities of the fragments. Table S2: Odor description of pure hemi-, mono-, and sesquiterpenes, which were given by at least three participants, with the number of mentions in parentheses and the average and the standard deviation of the intensity. Table S3: *D*-values and odor thresholds according to Teranishi et al. (*OT*(*Teranishi*)), odor thresholds determined with the individual odor thresholds (*OT**(*individual*)) of the three participants in the GC-O measurements. Table S4: Compounds with their respective odor descriptions determined by means of GC-O and the odor thresholds of the three participants in the air, * = mixture of (*E*) and (*Z*) isomers.

## Sample Availability

Samples of the compounds are not available from the authors.

## Abbreviations

DMAPP	dimethylallyl pyrophosphate
DT	detection threshold
GC-O	gas chromatography-olfactometry
*ee*	enantiomeric excess
FPP	farnesyl pyrophosphate
IPP	isopentenyl pyrophosphate
IS	internal standard
n.a.	not available
ODP	olfactory detection port
OT	odor threshold
OT_W_	odor threshold in water
OT_air_	odor threshold in air
n.d.	not determined
RT	recognition threshold
SAM	S-adenosyl methionine
