# Supplementary material for: Odor Characteristics of Novel Non-Canonical Terpenes
Source: Molecules. 2022 Jun 14;27(12):3827. doi: 10.3390/molecules27123827 (PMC9230113; doi:10.3390/molecules27123827)
Supplement: Supplementary file 1 [file molecules-27-03827-s001.zip › molecules-1759055-supplementary.pdf]

Article

Odor Characteristics of Novel Non-Canonical Terpenes

Svenja Sommer [1], Leon M. Lang [1], Laura Drummond [2], Markus Buchhaupt [2], Marco A. Fraatz [1] and Holger Zorn [1,3,*]

[1] Institute of Food Chemistry and Food Biotechnology, Justus Liebig University Giessen, Heinrich-Buff-Ring 17, 35392 Giessen, Germany; svenja.sommer@lcb.chemie.uni-giessen.de
[2] Microbial Biotechnology, DECHEMA- Forschungsinstitut, Theodor-Heuss-Allee 25, 60486 Frankfurt, Germany; markus.buchaupt@dechema.de
[3] Fraunhofer Institute for Molecular Biology and Applied Ecology, Ohlebergsweg 12, 35392 Giessen, Germany; holger.zorn@ime.fraunhofer.de
* Correspondence: holger.zorn@uni-giessen.de; Tel.: +49-(0)-641-99-4900

Supplementary Materials

Table S1. Gas chromatography-mass spectra of unpublished compounds with the relative intensities of the fragments.

Compounds	Fragments with relative intensities
4-methyl-3-carene	107 (100%), 150 (47%), 91 (43%), 135 (32%), 93 (26%), 94 (24%), 105 (23%), 79 (20%), 106 (14%), 108 (12%)
4-methylgeraniol	69 (100%), 137 (72%), 43 (54%), 98 (52%), 41 (49%), 81 (44%), 82 (38%), 67 (37%), 107 (33%), 125 (33%)
8-methylgeraniol	55 (100%), 83 (68%), 137 (37%), 93 (36%), 84 (24%), 121 (22%), 67 (22%), 41 (21%), 82 (15%), 81 (14%)
2-methylisoprenol	71 (100%), 67 (96%), 41 (91%), 69 (77%), 55 (51%), 82 (42%), 100 (19%), 42 (18%), 57 (16%), 68 (15%)
2,4-dimethylisoprenol (isomer A)	83 (100%), 55 (71%), 114 (38%), 41 (18%), 84 (13%), 81 (12%), 43 (12%), 67 (11%), 96 (11%), 69 (7%)
2,4-dimethylisoprenol (isomer B)	83 (100%), 55 (67%), 114 (46%), 41 (18%), 81 (17%), 67 (14%), 43 (12%), 84 (11%), 96 (10%), 69 (7%)
2,5-dimethylisoprenol	83 (100%), 55 (81%), 81 (43%), 69 (32%), 41 (26%), 67 (26%), 96 (24%), 84 (19%), 82 (12%), 56 (11%)
5-methylisoprenol	67 (100%), 822 (59%), 55 (41%), 41 (26%), 89 (20%), 70 (17%), 81 (13%), 42 (12%), 54 (12%), 53 (9%)
4-methylnerol	69 (100%), 41 (56%), 83 (55%), 98 (54%), 107 (44%), 67 (40%), 82 (39%), 81 (39%), 43 (38%), 91 (37%)
2,4-dimethylprenol (isomer A)	85 (100%), 96 (78%), 81 (56%), 43 (31%), 67 (24%), 55 (23%), 41 (22%), 79 (17%), 114 (15%), 57 (11%)
2,4-dimethylprenol (isomer B)	85 (100%), 114 (40%), 81 (37%), 43 (24%), 96 (19%), 41 (17%), 55 (17%), 67 (17%), 79 (13%), 57 (8%)
2-methylprenol	85 (100%), 100 (52%), 67 (46%), 41 (29%), 82 (29%), 43 (21%), 65 (10%), 57 (9%), 55 (8%), 79 (7%)
4,5-dimethylprenol	85 (100%), 67 (53%), 43 (28%), 96 (27%), 55 (27%), 41 (23%), 57 (21%), 81 (19%), 79 (10%), 53 (8%)

Table S2. Odor description of pure hemi-, mono-, and sesquiterpenes, which were given by at least three participants, with the number of mentions in brackets and the average and the standard deviation of the intensity (0 = no odor, 5 = very intensive), (*n* = 15).

Compounds	Odor impression	Intensity
(*S*)-borneol	earthy (11), moldy (9), woody (4), moss (4), forest (3)	3.8 ± 1.4
(*S*/*R*)-camphene	resinous (7), woody (5), herbal (3), coniferous forest (6)	2.6 ± 1.1
(*R*)-camphene	resinous (7), coniferous forest (7)	1.4 ± 1.4
(*S*)-3-carene	resinous (10), coniferous forest (7), herbal (4), ethereal (3)	3.5 ± 0.8
(*R*)-citronellol	flowery (7), citrus (6), lemon (6), fresh (3)	3.1 ± 1.5
(*S*)-citronellol	citrus (8), flowery (7), sweetish (6), resinous (5), lemon peel (4), ethereal (3), rose (3)	3.8 ± 1.4
(*E*,*E*)-farnesol	sweetish (4)	0.4 ± 0.5
(*R*)-α-fenchol	earthy (11), moldy (8), moss (5), forest (3), woody (3)	4.7 ± 0.6
geraniol	flowery (8), citrus (7)	2.6 ± 1.5
isoprenol	sweetish (4), fruity (4), varnish (5), citrus (4), flowery (3), terpene (3)	3.3 ± 0.8
(*R*)-limonene	resinous (7), citrus (6), terpene (3)	3.3 ± 0.7
(*S*)-limonene	resinous (8), green (4), citrus (4), coniferous forest (4), mango (3)	4.1 ± 0.9
linalool	flowery (8), blueberry (7), sweetish (7), citrus (5)	3.3 ± 0.9
(*R*)-linalool	fruity (4), flowery (3), resinous (3), blueberry (3), mint (3), green (3)	3.9 ± 0.8
nerol	resinous (8), citrus (7), flowery (4), ethereal (4), mango (3), herbal (3), orange (3), fruity (3), fresh (3), terpene (3)	3.6 ± 1.0
prenol	resinous (7), herbal (4), terpene (4), woody (3), forest (3)	2.7 ± 1.0
(*S*)-α-terpineol	-[#]	0.6 ± 0.8

[#] No impression was named by ≥ 3 participants.

Table S3. *D*-values and odor thresholds according to Teranishi *et al.* (*OT(Teranishi)*), odor thresholds determined with the individual odor thresholds (*OT*(individual)*) of the three participants in GC-O measurements, ˜ = determined with increased initial concentration (2300 mg L^{-1} (*S*)-3-carene and 1-methylcamphene 3650 mg L^{-1}), * = mixture of (*E*) and (*Z*)-isomer.

Compounds	*D-values*			OT(Teranishi) / ng L^{-1}			OT*(individual) / ng L-1		
	D_1	D_2	D_3	OT_1	OT_2	OT_3	OT^*_1	OT^*_2	OT^*_3
(*S*)-1-methylcamphene	2	1	4˜	1000	2100	1000	3100 ±1000	3100 ± 1000	6200 ± 2100
(*R*)-camphene	2	4	1	1300	660	660	3900 ±1300	980 ± 330	3900 ±1300
(*R*/*S*)-camphene	2	4	1	1100	570	570	3400 ± 1100	840 ± 280	3200 ± 1100
4-methyl-3-carene	2	8	1	920	230	460	2700 ± 910	340 ± 110	2700 ± 910
(*S*)-3-carene	1	4	4˜	2600	650	650	7800 ± 2600	970 ± 320	3900 ± 1300
2-methylcitronellol A	64	32	128	6.9	14	0.86	21 ± 6.8	21 ± 6.8	5.1 ± 1.7
2-methylcitronellol B	64	16	32	3.9	16	1.9	12 ± 3.8	23 ± 7.7	12 ± 3.8
(*R*)-citronellol	16	8	16	22	44	5.5	65 ± 22	65 ± 22	32 ± 11
(*S*)-citronellol	64	8	16	5.6	45	5.6	17 ± 5.5	66 ± 22	33 ± 11
(*S*)-2-methyl-*α*-fenchol	32	64	16	5.4	2.7	2.7	16 ± 5.3	4.0 ± 1.3	16 ± 5.3
(*R*)-*α*-fenchol	8	16	2	22	11	22	65 ± 22	16 ± 5.4	130 ± 43
2-methylnerol	4	8	2	86	43	43	260 ± 85	64 ± 21	260 ± 85
2-methylgeraniol	64	32	2048	5.4	11	0.043	16 ± 5.3	16 ± 5.3	0.25 ± 0.083
4-methylnerol	4	1	2	24	97	12	72 ± 24	143 ± 48	72 ± 24
4-methylgeraniol	16	64	256	37	9.3	0.58	110 ± 37	14 ± 4.6	3.4 ± 1.1
8-methylgeraniol*	64	8	32	11	86	5.4	32 ± 11	128 ± 43	32 ± 11
geraniol	32	16	128	5.6	11.2	0.35	17 ± 5.6	17 ± 5.6	2.1 ± 0.70
nerol	2	128	64	180	2.8	1.4	530 ± 180	4.1 ± 1.4	8.3 ± 2.8
2-methyllimonene	4	8	8	350	170	43	1000 ± 340	260 ± 86	260 ± 86
(*R*)-limonene	32	64	4	88	44	180	260 ± 87	65 ± 22	1000 ± 350
(*S*)-limonene	8	32	16	180	44	22	520 ± 170	65 ± 21	130 ± 44
2-methyllinalool	128	128	16	1.4	1.4	2.7	4.0 ± 1.3	2.0 ± 0.67	16 ± 5.3
(*R*)-linalool	1024	2048	1024	0.17	0.084	0.042	0.50 ± 0.17	0.13 ± 0.042	0.25 ± 0.083
(2*E*)-decenal	64	128	64		2.7		8.0 ± 2.7	4.0 ± 1.3	16 ± 5.3

Table S4. Compounds with their respective odor descriptions determined by means of GC-O and the odor thresholds of the three participants in air, * = mixture of (*E*)- and (*Z*)-isomer.

Compounds	Odor impression
(*S*)-1-methylcamphene	resinous, coniferous forest, sweetish
(*R*)-camphene	resinous, coniferous forest, etheric, sweetish
(*R*/*S*)-camphene	resinous, earthy, plastic, green, sweetish
4-methyl-3-carene	resinous, musty, citrus, green, sweetish
(*S*)-3-carene	resinous, plastic, herbal, terpene-like
2-methylcitronellol A	citrus, lemon, sweetish, soapy
2-methylcitronellol B	citrus, lemon, sweetish, soapy
(*R*)-citronellol	lemon, citrus, sweetish
(*S*)-citronellol	lemon, citrus, sweetish
(*S*)-2-methyl-α-fenchol	mold, earthy, musty
(*R*)-α-fenchol	mold, earthy, musty
2-methylnerol	citrus, flowery, sweetish, orange, fresh
2-methylgeraniol	citrus, flowery, sweetish, geranium
4-methylnerol	fruity, flowery, citrus, green
4-methylgeraniol	citrus, flowery, fruity, orange, rose
8-methylgeraniol*	flowery, rose, citrus, waxy
geraniol	flowery, rose, citrus, waxy
nerol	citrus, flowery, terpene-like, resinous
2-methyllimonene	resinous, orange, coniferous forest, fungal
(*R*)-limonene	resinous, coniferous forest, citrus, terpene-like
(*S*)-limonene	resinous, citrus, orange, mango, peppery
2-methyllinalool	flowery, sweetish, citrus, woody
(*R*)-linalool	citrus, lavender, blueberry, sweetish
